# Exploring the mechanism of amebic trogocytosis: the role of amebic lysosomes

**DOI:** 10.15698/mic2018.01.606

**Published:** 2017-12-06

**Authors:** Allissia A. Gilmartin, William A. Petri, Jr

**Affiliations:** 1 Department of Microbiology, Immunology and Cancer Biology, University of Virginia, Charlottesville, VA, USA; 2 Department of Medicine, University of Virginia, Charlottesville, VA, USA.; 3 Department of Pathology, University of Virginia, Charlottesville, VA, USA.

**Keywords:** amebiasis, entamoeba, trogocytosis, cytotoxicity

Trogocytosis, the intracellular transfer of fragments of cell material (including intact proteins but not whole cells) has been implicated in both microbial pathogenesis and the host defense against pathogens. Although trogocytosis has been observed in a number of organisms, from humans to amebae, the process remains poorly understood. *Entamoeba histolytica* is a protozoan parasite and human pathogen that kills human cells in a nibbling-like process termed amebic trogocytosis. We have recently found that amebic lysosomes play a key role in amebic trogocytosis and cell killing. Impairing lysosome acidification with pharmacological inhibitors dramatically decreased amebic trogocytosis and cell killing. Our data suggest that these inhibitors do not block the ingestion of an initial fragment, but rather prevent continued trogocytosis and the ingestion of multiple fragments. These findings raise a number of interesting biological questions, notably how do lysosomes function in amebic trogocytosis? A deeper understanding of the role of lysosomes in trogocytosis could provide interesting insights into trogocytosis as a fundamental biological process.

*Entamoeba histolytica* causes potentially fatal diarrhea, dysentery and liver abscesses in humans 1. Tissue destruction is the hallmark of invasive *E. histolytica *infection, manifesting as massive intestinal ulceration and abscesses at other sites. This tissue destruction is likely driven by amebic trogocytosis 2. Amebic trogocytosis begins with the attachment of the parasite to a host cell, followed by ingestion of fragments of the host cell. The fragments appear to be surrounded by two membranes, an outer membrane derived from the amebic plasma membrane and an inner layer derived from the host membrane. The fragments were shown to contain host cell membrane, cytoplasm, and mitochondria. The parasite continues to ingest fragments of the host cell until the host cell eventually dies. It appears that this continued ingestion of multiple fragments is necessary for cell killing and pathogenesis 2. Unfortunately, little is known about the mechanism of amebic trogocytosis.

Trogocytosis has been observed in a number of other organisms. In humans, trogocytosis has been observed in lymphocytes, including T-, B-, NK and dendritic cells. In lymphocytes, trogocytosis has been implicated in cell-cell communication (with ingestion of the T cell receptor complexed with HLA from antigen presenting cells implicated in sustained activation of T cells) and defense against pathogens 3,4. The process is distinguished from other methods of intracellular transfer, such as phagocytosis, by the transfer of pieces of cell material (including intact proteins but not whole cells), the requirement for close cell-cell contact, and the rapid rate of uptake (within minutes), all of which are reminiscent of what we observe in amebic trogocytosis 3. In T- and NK-cells, trogocytosis is a metabolically active process that requires signaling in the acceptor cell, and modulation of both the actin cytoskeleton and intracellular Ca^2+ ^5. Small GTPases and phosphatidylinositide 2-kinase (PI3K) have also been identified as key players in T-cell trogocytosis 5. A process referred to as trogocytosis has been observed in the free-living amoeba *Naegleria fowleri, *a human parasite that may use this process to destroy host cells, but again the mechanism is unknown 6,7. A trogocytosis-like process observed in *Plasmodium falciparum*-infected red blood cells has been implicated in the pathogenesis of cerebral malaria 6,7,8. Trogocytosis also plays a role in the spread of the intracellular bacteria *Francisella tularensis *9.

Early research indicated that inhibiting amebic lysosomal acidification impaired amebic killing of human cell 10. Interestingly, studies comparing *E. histolytica *with the less-pathogenic species *Entamoeba dispar* have noted that acidification of the phagosomes takes significantly longer in *E. dispar *and does not reach the same level of acidification, suggesting a role for lysosomes in the pathogenesis of amebiasis 11. In our recent work, we found that inhibition of lysosome acidification by either a weak base or a vacuolar -ATPase inhibitor significantly decreased amebic trogocytosis and cell killing 12. We observed that interference with acidification does not impact the initiation of amebic trogocytosis, but rather impairs continued ingestion and cell killing. Treatment with acidification inhibitors also decreased phagocytosis, suggesting that lysosomes play a crucial role in this process as well. Importantly, fluid-phase endocytosis was not affected, indicating that these drugs do not simply block all endocytic processes 12.

**Figure 1:**
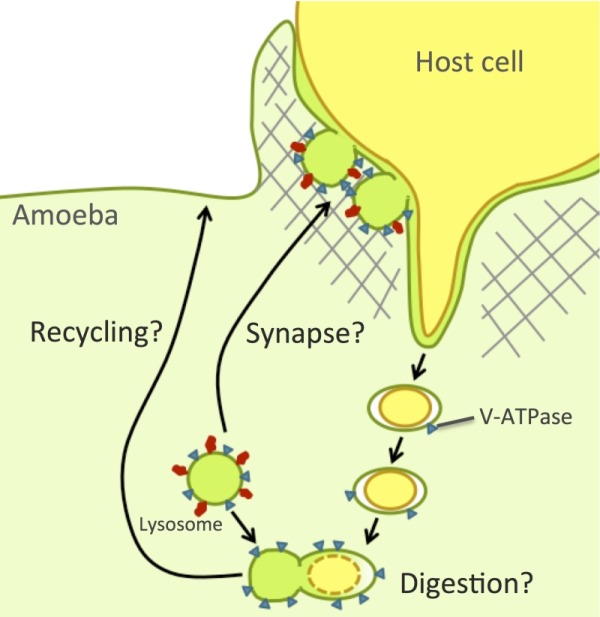
FIGURE 1: Model of possible roles for amebic lysosomes in amebic trogocytosis and cell killing. Amebic lysosomes might be required for rapid degradation of the ingested fragments, for efficient recycling of ingested receptors and membrane, or for the rapid formation of an acidified synapse at the site of ameba-host interaction. Blue triangles vesicles indicate V-ATPases. Red ovals indicate amebic Rab7B.

There are several possible roles that lysosomes might play in amebic trogocytosis (Figure 1): lysosomes might be required for efficient degradation of the ingested fragments, for rapid recycling of ingested receptors and membrane, or for the rapid formation of an acidified synapse at the site of ameba-host interaction. During amebic trogocytosis, parasites ingest numerous host cell fragments, which must be degraded. In eukaryotes, it is well established that lysosomes are crucial for the turnover of ingested material. Breakdown of material in the lysosome requires lysosomal enzymes, many of which are pH-dependent. Weak bases, such as the ammonium chloride used in our studies, raise the pH of amebic lysosomes, which would impair the function of pH-dependent lysosomal enzymes 10. Indeed, previous work has shown that concanamycin A-treated parasites fail to acidify their phagosomes to normal levels and are impaired in their ability to degrade phagocytosed *Leishmania *11*.* Thus it is possible that lysosomes are crucial for continued amebic trogocytosis and cell killing because they are required for efficient degradation of the ingested host fragments.

Previous work has shown that trogocytosed fragments are surrounded by amebic membrane, likely derived from the amebic plasma membrane 2. Thus, it is likely that the amebae internalize plasma membrane and surface receptors as they ingest fragments. Our data show that interference with acidification blocks receptor-dependent processes - both amebic trogocytosis and phagocytosis - but does not impair a receptor-independent process, fluid-phase endocytosis 12. Together these data suggest that rapid recycling of membrane and receptors, facilitated by the amebic lysosomes, may be required for continued amebic trogocytosis and, thus, cell killing.

It has been suggested that amebic lysosomes may form an acidified synapse at the site of host cell attachment similar to the synapse created by mammalian osteoclasts, which secrete lysosomes into an acidified synapse during bone matrix degradation 13. Live confocal microscopy and electron microscopy have both shown a massive accumulation of actin and exclusion of vesicles at the site of active host fragment ingestion, making this hypothesis less likely 2. However, it is possible that amebic lysosomes fuse at the ameba-host interface after attachment but before active ingestion begins.

In conclusion, this work sheds new light on an observation, first made 30 years ago, that weak bases inhibit amebic killing of human cells, by demonstrating that acid vesicle neutralization acts through the inhibition of trogocytosis. This work raises several interesting questions and further investigation will contribute to a better understanding of the nature of trogocytosis as a fundamental biological process.
